# Molecular Mechanisms of Liver Injury and Hepatocarcinogenesis: Focusing on the Role of Stress-Activated MAPK

**DOI:** 10.1155/2012/172894

**Published:** 2012-05-14

**Authors:** Hayato Nakagawa, Shin Maeda

**Affiliations:** ^1^Department of Gastroenterology, University of Tokyo, 7-3-1 Hongo, Bunkyo-ku, Tokyo 113-8655, Japan; ^2^Laboratory of Gene Regulation and Signal Transduction, Departments of Pharmacology and Pathology, School of Medicine, University of California, La Jolla, CA 92093, USA; ^3^Department of Gastroenterology, Yokohama City University, 3-9 Fukuura, Kanazawa-ku, Yokohama 236-0004, Japan

## Abstract

Hepatocellular carcinoma (HCC) is the third most common cause of cancer mortality. Short-term prognosis of patients with HCC has improved recently due to advances in early diagnosis and treatment, but long-term prognosis is still unsatisfactory. Therefore, obtaining a further understanding of the molecular carcinogenic mechanisms and the unique pathogenic biology of HCC is important. The most characteristic process in hepatocarcinogenesis is underlying chronic liver injury, which leads to repeated cycles of hepatocyte death, inflammation, and compensatory proliferation and subsequently provides a mitogenic and mutagenic environment leading to the development of HCC. Recent *in vivo* studies have shown that the stress-activated mitogen-activated protein kinase (MAPK) cascade converging on c-Jun NH_2_-terminal kinase (JNK) and p38 plays a central role in these processes, and it has attracted considerable attention as a therapeutic target. However, JNK and p38 have complex functions and a wide range of cellular effects. In addition, crosstalk with each other and the nuclear factor-kappaB pathway further complicate these functions. A full understanding is essential to bring these observations into clinical settings. In this paper, we discuss the latest findings regarding the mechanisms of liver injury and hepatocarcinogenesis focusing on the role of the stress-activated MAPK pathway.

## 1. Introduction

 Hepatocellular carcinoma (HCC) is the fifth most common cancer and the third most common cause of cancer mortality worldwide [1]. The age-adjusted incidence rate of HCC varies geographically, with high rates in East Asia and moderate rates in Europe, North/South America, and Oceania. In addition, a definite increase in HCC incidence has recently been reported in Europe and North America [2].

 Although the etiology of background liver disease varies geographically, chronic hepatitis B virus (HBV) or hepatitis C virus (HCV) infection is the main cause of HCC in most areas. Other major etiologies include alcoholic hepatitis, hemochromatosis, and nonalcoholic steatohepatitis (NASH) [1, 3]. Accumulating evidence indicates that a sustained inflammatory reaction in the liver is the major contributing factor to the development of HCC. For example, in chronic hepatitis C, the host immune responses to HCV are often not strong enough to completely clear the infection, resulting in chronic stimulation of an antigen-specific immune response [4]. Hepatocyte damage is induced by the continued expression of cytokines and recruitment of activated inflammatory cells to the liver, which is followed by hepatocyte regeneration. This persistent cycle of necroinflammation and hepatocyte regeneration is thought to provide a mitogenic and mutagenic environment leading to the development of HCC [5–7].

 Effective treatments for HCC include surgical resection, percutaneous ablation, and liver transplantation. Although liver transplantation results in a better survival rate [8], its application is limited by the scarcity of donor organs. Surgical resection and percutaneous ablation also provide a high rate of complete responses [9, 10], but long-term prognosis is not satisfactory, as indicated by the low overall survival of 22%–35% at 10 years [2]. This is due to frequent recurrence and lack of effective treatment for advanced HCC. Transarterial chemoembolization, radiotherapy, and conventional chemotherapy have been performed for advanced HCC, but their efficacies are limited [11].

 Recently, molecular-targeted therapy was developed for the treatment of HCC. Sorafenib is a small molecule multikinase inhibitor targeting the Raf serine/threonine kinases, vascular endothelial growth factor receptor (VEGFR) and platelet-derived growth factor receptor (PDGFR) pathway. In the SHARP (Sorafenib HCC Assessment Randomized Protocol) trial, sorafenib significantly prolonged median overall survival compared to placebo (10.7 *versus *7.9 months, resp.) [12]. However, median survival was not satisfactory even in the sorafenib group. Although other molecular-targeting agents are currently under investigation, only limited improvements in survival benefit have been reported [13].

 Eradication of the cause of chronic inflammation, such as hepatitis viruses, is the most beneficial means of preventing HCC [14]. Recent advances in antiviral treatment have made the eradication of HCV or significant suppression of HBV replication possible [15–17]. However, these therapies are not effective for all patients, and the development of HCC will not be prevented completely, especially in patients with advanced fibrosis. In addition, in patients with HCC based on metabolic syndrome including NASH, the incidence rate of which is increasing, dietary modifications and exercise are fundamental, but these approaches are sometimes ineffective and genetic background may also be important in such cases. Furthermore, once HCC has developed, the recurrence rate does not decline with time regardless of background liver disease, suggesting that most cases of late-phase recurrence are due to metachronous multicentric carcinogenesis [11]. Thus, gaining further understanding of the molecular mechanisms of carcinogenesis in HCC is important to discover new molecular targets, especially for preventing the occurrence or recurrence of HCC and systemic therapy for patients in the advanced stage.

 Several molecular pathways have been reported to play important roles in hepatocarcinogenesis. As mentioned above, inflammation-mediated liver injury and compensatory hepatocyte proliferation are indispensable components during HCC development. Recent high-quality *in vivo* studies have shown that stress-activated mitogen-activated protein kinase (MAPK) signaling converging on c-Jun NH_2_-terminal kinase (JNK) and p38 plays a central role in these processes, and it has attracted considerable attention as a therapeutic target [18–24]. In this paper, we discuss the mechanisms of liver injury and hepatocarcinogenesis focusing on the role of the stress-activated MAPK pathway.

## 2. General Role of JNK in the Liver

### 2.1. Stress-Activated MAPK

**Figure 1:**
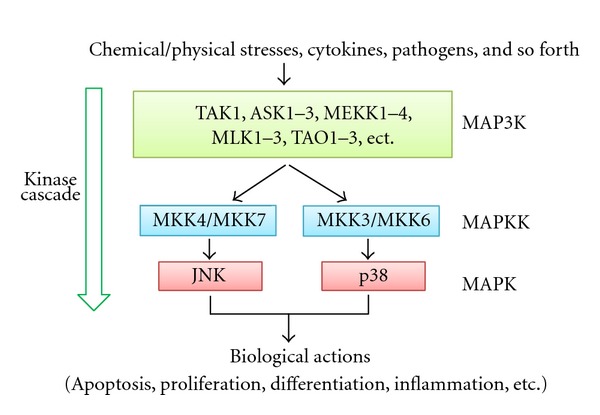
Stress-activated MAPK signaling pathway. This pathway consists of three classes of protein kinase—MAP3K, MAPKK, and MAPK—and converges on JNK and p38 through a kinase cascade. Only representative molecules are shown in MAP3K.

 MAPK cascades are signaling systems that transmit stimuli from outside the cell to the nucleus [25, 26]. Three major MAPK cascades have been characterized in detail, converging on ERKs, JNKs, and p38 MAPKs; each consists of three classes of serine/threonine kinases, MAPK, MAPK kinase (MAPKK), and MPKK kinase (MAP3K). MAP3K phosphorylates and thereby activates MAPKK, and activated MAPKK in turn phosphorylates and activates MAPK (Figure 1). Among the three MAPKs, ERKs are activated by various cytokines and growth factors and play a central role in cell growth and differentiation. JNKs and p38 MAPKs, also called stress-activated MAPKs, are preferentially activated by proinflammatory cytokines, such as tumor necrosis factor *α* (TNF*α*) and environmental and genotoxic stresses. After activation, stress-activated MAPKs phosphorylate specific serine/threonine residues of target substrates and exert a variety of cellular functions, such as cell death, survival, proliferation, migration, and inflammation.

### 2.2. JNK Signaling

 JNK has three isoforms (JNK1, JNK2, and JNK3) encoded by three different genes. JNK1 and JNK2 are expressed in almost all organs, including the liver, whereas JNK3 is a neuronal-specific isoform [27]. JNK is phosphorylated and activated by two MAPKKs, MKK4, and MKK7. Activated JNK phosphorylates and thereby activates c-Jun, JunD, ATF, and other transcription factors, which are involved in the formation and activation of the AP-1 complex [28]. In addition, JNK also modulates the function of other proteins by phosphorylation, such as Bcl-2 family members, which are closely related to apoptotic cell death factors [29]. Furthermore, although JNK1 and JNK2 isoforms play redundant roles in many physiological processes, they also have distinct biological activities in some situations [30]. For example, JNK1-deficient CD8^+^ T cells are unable to undergo antigen-stimulated expansion, whereas JNK2-deficient CD8^+^ T cells are hyperproliferative [31]. Therefore, the role of JNK in controlling diverse cellular functions, such as cell proliferation, differentiation, and apoptosis, is based on the phosphorylation and functional modification of downstream molecular targets in a stimulus- and cell-type-dependent manner.

### 2.3. Role of JNK in TNF*α*-Mediated Liver Injury

 Underlying liver injury, which leads to repeated cycles of hepatocyte death, inflammation, and compensatory proliferation, is an indispensable factor in the development of most liver cancers. Therefore, determining the mechanism of liver injury is a basic requirement for understanding hepatocarcinogenesis.

 The proinflammatory cytokine TNF*α* plays a pivotal role in various inflammatory liver diseases [32]. Serum and hepatic TNF*α* levels are increased in patients with acute and chronic viral hepatitis, alcoholic hepatitis, and NASH [33–36]. Although TNF*α* can kill hepatocytes during such liver diseases, the healthy liver usually has defense systems against TNF*α*-induced cell death. In fact, TNF*α* stimulates proliferation rather than death through DNA synthesis in the normal hepatocyte, which is required for liver regeneration after partial hepatectomy [37, 38]. However, once antiapoptotic response is inhibited, TNF*α* signaling is converted from proliferation to apoptosis. Thus, TNF*α* has the capacity to induce hepatocyte death as well as hepatocyte proliferation, and plays a crucial role in preserving liver homeostasis [39]. Recent studies have uncovered the important role of the cross talk between JNK and nuclear factor-kappaB (NF-*κ*B) pathways in these processes, as described later.

**Figure 2:**
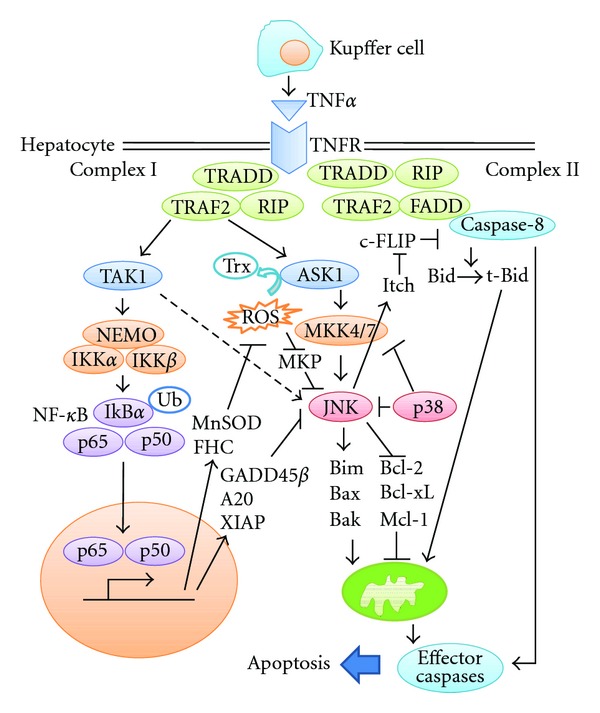
Signaling pathways of TNF*α*-induced hepatocyte death. After binding of TNF*α* to TNF-R1, complexes I and II are formed. As the amount of caspase-8 activation in complex II is not sufficient to induce cell death of hepatocytes, activation of the mitochondrial apoptotic pathway is essential. Activated caspase-8 initiates the mitochondrial apoptotic pathway by cleaving Bid to tBid. In addition, JNK can also activate the mitochondrial apoptotic pathway though modulation of Bcl-2 family members or degradation of c-FLIP. These pathways cooperate to induce cell death, while NF-*κ*B and p38 inhibit TNF*α*-induced hepatocyte death by suppressing JNK activation through various mechanisms.

 TNF*α* is produced mainly by Kupffer cells in the liver and exerts biological effects by binding to two plasma membrane receptors, TNF-receptor 1 (TNF-R1) and TNF-R2 [40]. The majority of the biological effects of TNF*α* are mediated by TNF-R1. Upon binding of TNF*α* to TNF-R1, the signaling molecule TNF-R-associated death domain (TRADD), TNF-R-associated factor 2 (TRAF2), and receptor interacting protein 1 (RIP1) are recruited to form the so-called complex I [41] (Figure 2). Assembly of this complex is essential to activate two key players, JNK and NF-*κ*B pathways.

 In addition to the formation of complex I, TNF*α* binding to TNF-R1 simultaneously recruits Fas-associated death domain (FADD) and caspase-8, thus forming another complex, the so-called complex II [41] (Figure 2). Here, caspase-8 is activated through autocatalytic cleavage and active caspase-8 is then able to proteolytically activate several effector caspases such as caspase-3. However, in the case of hepatocytes, the amount of active caspase-8 generated by complex II is too small for activation of effector caspases, requiring an amplification loop through mitochondria [42]. Caspase-8 cleaves Bid to a truncated form, tBid, which translocates to the mitochondria, causing activation of Bak/Bax and subsequent activation of the mitochondrial apoptotic pathway. This mitochondrial pathway involves the release of numerous apoptogenic factors from the mitochondrial intermembrane space into the cytosol, including cytochrome *c*, which activates effector caspases and then induces cell death and amplification of caspase-8 activation.

 The importance of JNK in TNF*α*-mediated liver injury has been confirmed by many *in vitro *and *in vivo* studies. In an *in vitro* study, TNF*α*-induced apoptosis was blocked by the small molecule JNK inhibitor SP600125 in mouse and rat primary hepatocytes [43]. *In vivo*, NF-*κ*B-deficient embryos, which normally die due to TNF*α*-mediated hepatocyte apoptosis, were shown to survive longer when the JNK1 gene was also disrupted [44]. In addition, JNK1 knockout (*JNK*1^−/−^) and JNK2 knockout (*JNK*2^−/−^) mice were resistant to concanavalin-A-(Con-A-) induced liver injury, which is primarily TNF*α* dependent [45]. In another study, *JNK*2^−/−^ mice, but not *JNK*1^−/−^, mice were shown to be resistant to lipopolysaccharide (LPS)/d-galactosamine (GalN)-induced liver injury, which is also a TNF*α*-dependent liver injury model [46]. A recent study showed that both *JNK*1^−/−^, and *JNK*2^−/−^, mice are resistant to LPS/GalN-induced liver injury, and JNK2 plays a more prominent role in TNF*α*-induced hepatocyte death [47]. Despite the discrepancies among studies concerning which of the JNK isoforms is more important in hepatocyte death, they all support the concept that JNK plays a key role in controlling TNF*α* signaling toward cell death.

 As TNF*α*-mediated hepatocyte death is also important for acute liver injury, JNK is considered to be a promising therapeutic target for acute liver injury. In fact, SP600125 and another JNK inhibitor D-JNKi, which is a peptide inhibitor of JNK, exert protective effects on LPS/GalN-induced fulminant hepatitis [44, 48].

### 2.4. Reactive Oxygen Species as Upstream Activators of JNK in TNF*α* Signaling

 TNF*α* binding to TNF-R1 leads to the formation of complex I and then activates a member of the MAP3K family, transforming growth factor *β*-activated kinase 1 (TAK1). Activated TAK1 activates MKK4 and MKK7, which leads to JNK activation [49]. However, transient activation of JNK is not sufficient to induce cell death. Reactive-oxygen-species-(ROS-) mediated prolongation of JNK activation is considered important for the proapoptotic function of JNK. This concept was supported by the higher levels of intracellular ROS observed in TNF*α*-treated cells and the observation that antioxidants, such as *N*-acetyl-l-cysteine which is precursor of glutathione and butylated hydroxyanisole which is synthetic antioxidant authorized as a food additive, can suppress TNF*α*-induced prolonged JNK activation and subsequent apoptosis [30].

 In TNF*α* signaling, two mechanisms have been proposed for ROS-mediated prolongation of JNK activation. One study indicated that ROS extended JNK activation by inactivating MAPK phosphatases (MKPs), which are essential for dephosphorylation of activated JNK [50]. TNF*α*-induced ROS oxidize critical Cys residues in the catalytic sites of various MKPs, leading to their inactivation, and ROS-mediated oxidation of MKP-1 rapidly leads to its degradation by the ubiquitin-proteasome pathway. Another study demonstrated an important role of apoptosis signal-regulating kinase 1 (ASK1), one of the major MAP3Ks, in the regulation of stress-activated MAPK [51]. TNF*α*-induced sustained activation of JNK is lost in *ASK*1^−/−^ embryonic fibroblasts, and *ASK*1^−/−^ cells are resistant to TNF*α*-induced apoptosis. While TAK1 is activated directly by TNF-R1-induced signaling, activation of ASK1 is secondary to the generation of ROS. The involvement of ASK1 in ROS-mediated JNK activation is based on links between ASK1 and thioredoxin (Trx), a reduction/oxidation regulatory protein [52]. In the absence of oxidative stress, Trx inhibits ASK1 kinase activity via direct binding to the N-terminal region of ASK1. Excess oxidative stress converts Trx to its oxidized form, resulting in its dissociation from ASK1 and subsequent ASK1 kinase activation. This system has been confirmed to function in the liver *in vivo* using an acetaminophen-induced liver injury model, which is a typical ROS-mediated liver injury model [53]. In addition, a recent study showed that *ASK*1^−/−^ mice are resistant to LPS/GalN-induced liver injury [54]. Furthermore, Trx transgenic mice are also resistant to LPS/GalN-induced liver injury [55]. Thus, TNF*α*-induced ROS prolongs JNK activity by blocking the inhibitory MKPs and simultaneously promoting the activation of ASK1, then shifting the balance from cell survival toward cell death.

 Viral proteins, including HCV core proteins, are also capable of ROS accumulation in hepatocytes, and ROS-mediated JNK activation in the liver is linked not only to liver disease but also to systemic disorders, such as insulin resistance and metabolic syndrome. Therefore, further elucidation of this process is very important [56–58].

### 2.5. Downstream Targets of JNK in TNF*α*-Induced Cell Death

 Several targets of JNK in TNF*α*-induced cell death have been proposed. The primary question is whether JNK is or is not involved in caspase-8 activation. JNK was initially suggested to induce caspase-8-independent cleavage of Bid at a site distinct from the Bid cleavage product jBid, which translocates to mitochondria and subsequently amplifies caspase-8 activation [59]. However, this finding has not been confirmed in an *in vivo* liver injury model. In the LPS/GalN-induced liver injury model, *JNK*2^−/−^ mice showed significant attenuation of caspase-8 activation and subsequent Bid cleavage, suggesting that JNK2 acts at the level of caspase-8 activation [46]. However, the detailed mechanism remains unclear and attenuated caspase-8 activation may be the indirect result of a lack of amplification by the mitochondrial release of proapoptotic factors. JNK may also be involved in caspase-8 activation through regulation of the caspase-8 inhibitor c-FLIP_L_ [44]. Prolonged activation of JNK1 leads to phosphorylation of Itch, thereby activating its ubiquitin ligase activity, which results in polyubiquitination and subsequent degradation of c-FLIP_L_. As c-FLIP_L_ competes with caspase-8 for binding to complex II and impairs caspase-8 activation, JNK1-mediated c-FLIP_L_ degradation results in full activation of caspase-8 and cell death. These studies support the suggestion that JNK is located upstream of caspase-8 activation.

 Other studies support the alternative suggestion that JNK plays an apoptotic role at a level farther downstream through the regulation of Bcl-2 family activation. Activated JNK has been shown to phosphorylate and inactivate the antiapoptotic proteins Bcl-2 and Bcl-xL [60–62]. Furthermore, activated JNK promotes Bax translocation to mitochondria through phosphorylation of 14-3-3, a cytoplasmic anchor of Bax. Phosphorylation of 14-3-3 leads to dissociation of Bax from this protein [63]. A more recent study showed that JNK contributes to TNF*α*-induced cell death through phosphorylation of the pro-apoptotic BH-3-only protein Bim, rather than Bid [64]. JNK-mediated Bim phosphorylation triggers the proapoptotic activity of Bim by causing its release from sequestration to the microtubular dynein motor complex and then activates the mitochondrial apoptotic pathway [65, 66]. These studies indicated that JNK acts in parallel and independent of caspase-8. Thus, although the downstream targets of JNK in TNF*α* signaling are not fully understood, most studies have demonstrated that JNK is required for activation of the mitochondrial apoptotic pathway.

### 2.6. Crosstalk between JNK and NF-*κ*B

 As prolonged JNK activation is closely related to cell death, systems for the regulation of JNK activity are mandatory for tissue homeostasis. The crosstalk between NF-*κ*B and JNK is considered to be a major determinant in the fate of hepatocytes, including TNF*α* signaling.

 NF-*κ*B is a transcription factor that consists of homodimers and heterodimers composed of five members, that is, p65 (RelA), c-Rel, RelB, p50, and p52 [67]. Each NF-*κ*B dimer has a different DNA-binding affinity and controls specific targets. Additionally, transcriptional activity of NF-*κ*B is regulated by transcription coactivators such as SRC family and corepressors such as HDAC family [68]. The p50/p65 heterodimer, the most abundant form of the NF-*κ*B, is regulated by so-called canonical pathway. Without stimulation, this heterodimer is bound to specific inhibitory factors, such as inhibitor of NF-*κ*B (I*κ*B) proteins in the cytoplasm. TNF*α* signaling complex I activates canonical NF-*κ*B pathway via the IKK complex, which consists of two catalytic subunits, IKK*α* and IKK*β*, and a regulatory component, IKK*γ*/NEMO (Figure 2). The IKK complex phosphorylates and subsequently induces degradation of I*κ*B. Once activated, NF-*κ*B dimers translocate into the nucleus and stimulate transcription of various genes, such as those encoding cytokines and antiapoptotic factors.

 Hepatocyte-specific IKK*β* or NEMO knockout mice are more sensitive to the hepatotoxic effects of injecting Con-A or LPS, respectively, suggesting a crucial role of NF-*κ*B activation in protection from TNF*α*-induced liver injury [45, 69]. Also note that the extent of TNF*α*-induced JNK activation is strengthened and prolonged in these mice.

 Several mechanisms have been proposed by which NF-*κ*B activation controls the level of JNK activation. At least three NF-*κ*B-dependent genes have been identified as potential candidates, that is, growth arrest DNA damage-inducible gene 45*β* (Gadd45*β*), X-linked inhibitor of apoptosis (XIAP), and the zinc finger protein A20 [70–76]. However, the precise implications and detailed mechanisms of action of these molecules in the regulation of TNF*α*-induced JNK activation and cell death remain unclear, especially *in vivo*. In addition to the expression of JNK inhibitory proteins, NF-*κ*B also inhibits JNK activation by controlling intracellular ROS levels. NF-*κ*B activation leads to expression of the antioxidants ferritin heavy chain (FHC) and manganese-dependent superoxide dismutase (MnSOD), both of which prevent accumulation of ROS, thus inhibiting prolongation of JNK activation [77, 78].

### 2.7. Crosstalk between JNK and Other MAPK Signaling Molecules

 p38*α* also plays an important role in inhibiting TNF*α*-induced JNK activation and liver injury. Hepatocyte-specific p38*α* knockout mice showed much stronger JNK activation after administration of LPS [79]. Although p38*α* ablation is not sufficient to induce hepatocyte death after LPS administration, hepatocyte-specific p38*α*, and IKK*β* double-knockout mice show severe liver injury, suggesting that p38*α* and IKK*β* act synergistically to protect the liver from TNF*α*-induced toxicity. Unlike in the case of NF-*κ*B, pretreatment with antioxidants could not reduce LPS-induced JNK activation in hepatocyte-specific p38*α* knockout mice. Furthermore, the levels of MKPs were similar between hepatocyte-specific p38*α* knockout mice and wild-type controls. Therefore, the detailed mechanisms of action of p38*α* in regulating TNF*α*-induced JNK activation and cell death remain unclear. However, hepatocyte-specific p38*α* knockout mice showed increased MKK4 activation, so targets of p38*α* are considered to be upstream of JNK, such as MAPKKs and MAP3Ks. Interestingly, not only MKK4 but also MKK3 and MKK6 activation, all of which are involved in p38 activation, are increased in hepatocyte-specific p38*α* knockout mice, suggesting that there might be negative feedback mechanism in p38 signaling pathway in the liver. In fact, p38 has been reported to negatively regulate upstream MAP3Ks such as TAK1 and MLK3 in other cells [80, 81].

 Two recent studies have introduced MAP3K TAK1 into the complex field of NF-*κ*B/JNK interaction in the liver [82, 83]. In TNF*α* signaling, TAK1 is recruited into complex I and phosphorylated through TRAFs. Then, phosphorylated TAK1 activates IKK and MKK4/7. Therefore, TAK1 can activate both NF-*κ*B and JNK, which play opposing roles in cell death. Inokuchi et al. reported that hepatocyte-specific TAK1 knockout mice generated by intercrossing TAK1 floxed mice with Alb-Cre mice showed spontaneous hepatocyte death, which was partially canceled by further genetic deletion of TNF-R1, suggesting that TAK1-deficient hepatocytes are sensitive to endogenous TNF*α* [82]. They also reported that hepatocyte-specific TAK1-deficient mice showed enhanced JNK activation *in vivo*. Primary hepatocytes derived from these mice also demonstrated enhanced cell death by TNF*α* stimulation, and not only NF-*κ*B activation but also JNK activation were inhibited *ex vivo*. Bettermann et al. reported that liver-specific TAK1 knockout mice generated by intercrossing TAK1 floxed mice with Alfp-Cre mice showed strong JNK activation, lack of NF-*κ*B activation, and massive hepatocyte apoptosis after LPS injection [83]. This enhanced JNK activation may occur through activation of other MAP3K TAO2. Furthermore, they showed that TAK1 is required for the prevention of cholangiocyte apoptosis. Thus, although the role of TAK1 in the regulation of TNF*α*-mediated JNK activation has not been fully elucidated, TAK1 plays an antiapoptotic role in the liver mainly through NF-*κ*B activation.

### 2.8. Implication of JNK in Other Types of Liver Injury

 JNK signaling has also been implicated in hepatocyte injury due to other causes, including lipotoxicity, ER stress, ischemia-reperfusion, and drug toxicity, such as from acetaminophen [53, 84–87]. Although the role of JNK in these types of hepatocyte death cannot be discussed here in detail due to space limitations, JNK is generally considered to play an important role in cell death induction through similar mechanisms as seen in TNF*α* signaling. Therefore, JNK is a possible candidate therapeutic target for various types of liver injury.

## 3. JNK in Hepatocarcinogenesis

### 3.1. General Function of JNK in Carcinogenesis

 Although this review has mainly discussed the pro-apoptotic function of JNK, this molecule has various functions in carcinogenesis. First, JNK plays an important role in cell proliferation. JNK is considered to be involved in cell cycle progression mostly through interaction with the transcription factor c-Jun, which is a well-established cell cycle regulator. A previous study using *JNK*1^−/−^ or *JNK*2^−/−^ fibroblasts showed that *JNK*2^−/−^ fibroblasts proliferate faster with increased c-Jun expression, whereas *JNK*1^−/−^ fibroblasts proliferate slower with decreased c-Jun expression, suggesting that JNK1 might play a major role in c-Jun-mediated cell cycle progression [88]. However, another study using fibroblasts from mice with a knockin mutation in the *JNK2* gene showed that JNK2 is also involved in c-jun expression and cellular proliferation [89]. Thus, in general, both JNK1 and JNK2 play important roles in cell cycle progression.

 In the liver, JNK1 has been shown to be a major player in cell cycle regulation using a partial hepatectomy model. Hui et al. reported that the number of Ki67-positive proliferating hepatocytes in *JNK*1^−/−^ mice was significantly reduced compared to wild-type controls after partial hepatectomy [20]. The impaired proliferation in *JNK*1^−/−^ mice was caused by increased expression of p21, a cell cycle inhibitor, and reduced expression of c-Myc, a negative regulator of p21. In contrast, *JNK*2^−/−^ mice showed similar hepatocyte proliferation rates to wild-type controls. Another study showed that administration of the pan-JNK inhibitor SP600125 inhibited hepatocyte proliferation after partial hepatectomy through reduced expression of cyclin D1 [90]. Thus, JNK plays an important role in hepatocyte proliferation *in vivo*.

 In addition to its roles in cell proliferation, JNK has been shown to also have various oncogenic functions. For example, the JNK pathway is involved in HCC cell migration and tumor invasion through matrix metalloproteinase (MMP) production [91]. A recent study showed that JNK is required for Ras-induced suppression of contact growth inhibition by regulating e-cadherin expression [92]. Furthermore, JNK facilitates cancer progression by interacting with other oncogenic pathways, such as Wnt/*β*-catenin signaling [93–95]. Thus, although JNK is generally considered to play oncogenic roles, it also plays roles in tumor suppression in some cases through p53 activation and induction of apoptosis [96–98].

### 3.2. Role of JNK in Hepatocarcinogenesis

 In general, induction of cell death acts as a tumor-suppressing function. However, this function does not always act as a tumor suppressor, but instead acts as a tumor promoter in the liver, because the loss of hepatic mass due to hepatocyte death activates the proliferation of residual hepatocytes that may provide a promoting environment for tumor formation. Recent *in vivo* studies strongly implicated JNK as a key regulator in this process.

**Figure 3:**
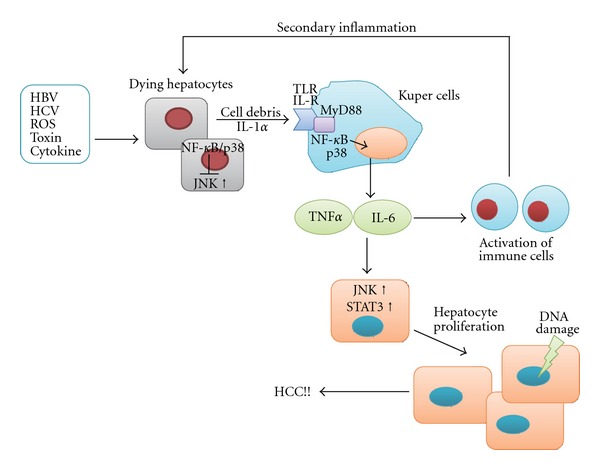
Current model of the role of stress-activated MAPK and NF-*κ*B in inflammation-mediated hepatocarcinogenesis. In chronic liver injury, hepatitis virus, ROS, toxins, and cytokines kill hepatocytes. JNK promotes cell death, while NF-*κ*B and p38 promote cell survival. Dying hepatocytes activate Kupffer cells via TLR or IL-1R. Activated Kupffer cells secrete cytokines, such as IL-6 and TNF*α*, through a mechanism mediated by NF-*κ*B and p38. Subsequently, these cytokines induce recruitment of activated inflammatory cells to the liver, which is followed by hepatocyte regeneration. JNK and STAT3 promote cytokine-mediated hepatocyte proliferation. This persistent cycle of necroinflammation and hepatocyte regeneration provides a mitogenic and mutagenic environment, leading to the development of HCC.

 Diethylnitrosamine (DEN) is a chemical carcinogen commonly used to induce HCC in rodents. Administration of DEN causes acute liver injury and DNA damage in the hepatocytes, and subsequently leads to inflammation, liver regeneration, and neoplastic lesions in the liver. A study using hepatocyte-specific IKK*β* knockout mice demonstrated a critical concept with regard to the role of cross talk between NF-*κ*B and JNK in hepatocytes in DEN-induced HCC [78] (Figure 3). Hepatocyte-specific knockout of IKK*β* markedly enhanced DEN-induced HCC. The ablation of hepatocyte IKK*β* induces greater DEN-induced JNK activation and greater cell death during the acute reaction to DEN administration. The absence of NF-*κ*B induces prolonged JNK activation by enhancing ROS production, and prolonged JNK activation plays a critical role in DEN-induced hepatocyte death, as seen in TNF*α*-mediated liver injury. Subsequently, the elevated hepatocyte death rate enhances compensatory proliferation. Thus, the hepatocyte-specific deletion of IKK*β* augments DEN-induced hepatocyte death, compensatory proliferation, and increased tumorigenesis, probably through enhanced JNK activation. Similar findings were obtained in mice lacking NEMO, the hepatocyte-specific deletion of which results in spontaneous liver damage, hepatosteatosis, fibrosis, and the development of HCC [69]. To further investigate the role of JNK in this process, *JNK*1^−/−^ mice were interbred with hepatocyte-specific IKK*β* knockout mice, and the double-knockout mice developed significantly fewer tumors compared to IKK*β* knockout mice [18]. In addition, *JNK*1^−/−^ mice showed a significant reduction of tumor development compared to wild-type controls. In addition to the involvement in DEN-induced acute liver injury, JNK1 has tumor-promoting functions by enhancing cancer cell proliferation and neovascularization through the increased expression of cyclin D1 and VEGF, respectively. Another group also reported the critical role of JNK1 in hepatocarcinogenesis using the DEN and phenobarbital-induced HCC model [20]. They showed that cancer cell proliferation decreases significantly in *JNK*1^−/−^ mice due to increased p21 expression and reduced c-Myc expression. In addition, pharmacological inhibition of JNK by D-JNKi reduced the growth of xenografted human HCC cells and chemically induced mouse liver cancers, suggesting that JNK is a promising therapeutic target for HCC. Their study, however, also showed that JNK2 is not involved in hepatocarcinogenesis. About 50%–60% of human HCC show strong activation of JNK1 compared to adjacent nontumor tissue, whereas JNK2 activation is similar between HCC and non-tumor tissue [20, 99]. These results suggest that JNK, especially JNK1, plays an important role in the development of HCC.

 JNK activation is also implicated in HCC based on viral hepatitis. *In vitro* studies showed that several HBV and HCV proteins can activate the JNK pathway [100, 101]. In addition, JNK-mediated enhanced cell proliferation is involved in hepatocarcinogenesis of HCV core protein transgenic mice and HBx protein transgenic mice, both of which spontaneously develop HCC [102, 103].

 However, a more recent study using conditional knockout mice of both JNK isoforms, JNK1 and JNK2, suggested that JNK may play dual roles in hepatocarcinogenesis [104]. In their study, JNK deficiency in hepatocytes increased DEN-induced HCC. In contrast, JNK deficiency in both hepatocytes and myeloid cells reduced hepatic inflammation and the development of HCC. Although the detailed mechanisms remain unclear, this study suggested that JNK in hepatocytes may act as a tumor suppressor, whereas that in myeloid cells may act as a tumor promoter in the development of HCC.

 JNK is also wellknown as a critical regulator of obesity and metabolic syndrome through inhibition of insulin signaling in the liver and muscle, and modulation of adipokine secretion from adipocytes [105, 106]. Recent studies have suggested that obesity-induced insulin resistance and dysregulation of adipokines play important roles in hepatocarcinogenesis [107–110]. As the incidence rate of this metabolic syndrome-associated hepatocarcinogenesis is likely to increase in the near future, investigating whether JNK activation also contributes to hepatocarcinogenesis under this condition is critical.

## 4. p38 and MAP3Ks in Hepatocarcinogenesis

### 4.1. p38 Signaling

 The p38 MAPK group consists of four members: p38*α*, p38*β*, p38*γ*, and p38*δ* [111]. p38*α* and p38*β* are universally expressed and closely related proteins that have overlapping functions. Whereas p38*α* is highly abundant in most cell types, p38*β* seems to be expressed at very low levels. p38*γ* and p38*δ* have more restricted expression patterns and are likely to have specialized functions [23]. Most of the published literature on p38 MAPKs refers to p38*α*, and we mainly discuss p38*α* in this paper. MKK3 and MKK6 are the primary upstream MAPKKs of p38, but MKK4 has also been shown to activate p38 in response to certain stimuli. p38 can also be activated independently of the MAP3K/MAPKK cascade by autophosphorylation [112]. Substrates of p38 include protein kinases, such as MAPKAP kinase 2 (MK2) and MK5, and transcription factors, such as ATF2, p53, and Mitf [113]. These diverse targets mediate the activated p38 signal to various types of cellular functions such as differentiation, apoptosis, cytokine production, and cell cycle control.

### 4.2. General Function of p38 in Carcinogenesis

 Although p38 was first recognized as a regulator of inflammatory cytokine production, recent studies uncovered a role of p38*α* as an essential inhibitor of cell proliferation. p38*α* negatively regulates cell cycle progression both at the G1/S and G2/M transitions by several mechanisms, including the downregulation of cyclins, upregulation of cyclin-dependent kinase (CDK) inhibitors, and modulation of the tumor suppressor p53 [114]. p38*α* also acts as an inhibitor of hepatocyte proliferation after partial hepatectomy, and inactivation of p38*α* is seen during liver regeneration [115, 116]. This effect of p38*α* is partially mediated by negative regulation of the JNK/c-Jun pathway [19].

 In addition to the suppression of cell proliferation, p38 exerts tumor-suppressing effects through oncogene-induced senescence (OIS), contact inhibition, and DNA damage responses [111]. In particular, recent studies demonstrated a major role of p38 in OIS caused by oncogenic Ras or its downstream effector Raf-1. The Ras-Raf-MEK-ERK signaling cascade increases the intracellular ROS level and subsequently activates the p38 pathway [117]. Activated p38 phosphorylates multiple residues on p53, leading to increased transcriptional activity of p53 and induction of p21 [111, 118]. Active p38 also induces the expression of p16 and p14/p19, which together with the p53-p21 cascade, cause senescence that serves as a tumor-suppressing defense mechanism [119]. Thus, a great deal of evidence supports a role of p38 as a tumor suppressor. However, p38 may also have oncogenic effects by facilitating cell invasion, inflammation, and angiogenesis [120, 121].

### 4.3. Role of p38 in Hepatocarcinogenesis

 The role of p38 in hepatocarcinogenesis has also been investigated using the DEN-induced mouse HCC model. Similar to hepatocyte-specific IKK*β* knockout mice, hepatocyte-specific p38*α* knockout mice showed enhanced DEN-induced ROS accumulation, JNK activation, liver damage, and subsequent hepatocyte proliferation, which resulted in enhanced carcinogenesis [21]. In addition, hepatocyte-specific p38*α* ablation leads to enhanced IL-1*α* release from necrotic hepatocytes. IL-1*α* can activate Kupffer cells, and activated Kupffer cells produce various cytokines, such as TNF*α*, IL-6, and hepatocyte growth factor, which promote proliferation of residual hepatocytes (Figure 3). The activation of p38 and NF-*κ*B in Kupffer cells plays an important role in this process because mice with p38*α* or IKK*β* knockout in both hepatocytes and myeloid cells, including Kupffer cells, showed markedly reduced hepatic expression of these cytokines after DEN challenge, resulting in reduced future HCC development [21, 78]. Thus, p38*α* and NF-*κ*B in the hepatocytes act as tumor suppressors by conferring protection against DEN-induced cell death, whereas p38*α* and NF-*κ*B in the Kupffer cells act as tumor promoters by enhancing cytokine production. Another study using hepatocyte-specific p38*α* knockout mice indicated its role in tumor suppression by focusing on the antiproliferative function at the advanced stage [19]. Ablation of p38*α* in the hepatocytes results in upregulation of the JNK/c-Jun pathway, which plays an important role in the increased proliferation of tumor cells.

 In human samples, the activity of the MKK6-p38 pathway is decreased in HCC tissues compared to adjacent non-tumor tissues [122]. In particular, its activity is significantly lower in larger HCC tissues, suggesting that the p38 pathway may play an antiproliferative role in human HCC.

### 4.4. Role of MAP3Ks in Hepatocarcinogenesis

 The evidence presented to date suggests that JNK generally acts as a tumor promoter and p38 generally acts as a tumor suppressor in hepatocarcinogenesis. However, as JNK and p38 have complex functions and modulate a wide range of cellular effect, some studies showed dual roles of these molecules in hepatocarcinogenesis. Furthermore, cross talk among JNK and p38, and molecules involved in other signaling pathways such as NF-*κ*B, further complicate their roles. Several recent studies have uncovered the roles of MAP3Ks in the regulation of stress-activated MAPK signaling in hepatocarcinogenesis.

 One player is TAK1, which can activate NF-*κ*B as well as JNK and p38 in response to the signaling of Toll-like receptors, IL-1 receptor, TNF receptor, and TGF-*β* receptor. As mentioned above, hepatocyte-specific TAK1 knockout mice show spontaneous hepatocyte death, which is partially mediated by endogenous TNF*α* [82, 83]. This spontaneous cell death subsequently causes compensatory hepatocyte proliferation, inflammation, and fibrosis. Furthermore, about 80%–90% of these mice spontaneously develop HCC at 9 months of age. These phenomena, which resembled the phenotype observed in hepatocyte-specific NF-*κ*B knockout mice, are considered to occur through attenuated NF-*κ*B activation and elevated JNK activation. The p38 pathway, however, seems not to be involved in this phenotype. Thus, TAK1 in the hepatocytes acts as a tumor suppressor mainly by regulating activation of the NF-*κ*B pathway. Unexpectedly, crossing hepatocyte-specific TAK1 knockout mice with NEMO knockout mice attenuates JNK activation and prevents hepatocyte death and the development of HCC, suggesting that NEMO has a tumor-promoting function in the setting of TAK1 deletion [83]. Furthermore, this function of NEMO is considered to be independent of NF-*κ*B.

**Figure 4:**
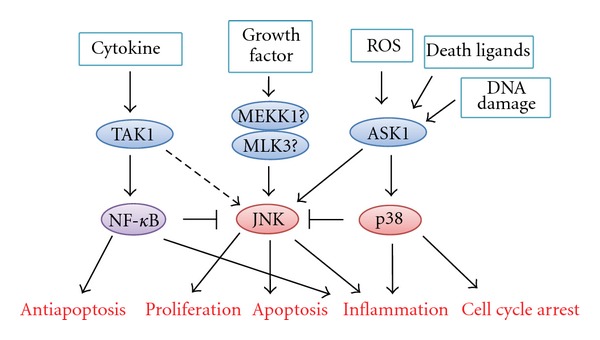
Roles of MAP3Ks in controlling the function of stress-activated MAPK and NF-*κ*B in hepatocarcinogenesis. TAK1 can activate NF-*κ*B and JNK, but TAK1 mainly regulates NF-*κ*B activation and acts as a tumor suppressor in hepatocarcinogenesis. ASK1 controls the tumor-suppressing function of JNK and p38 through apoptosis induction and DNA damage response, and thus acts as a tumor suppressor. MEKK1 and MLK3 may be involved in JNK-mediated tumor cell proliferation in hepatocarcinogenesis, but no supporting *in vivo* evidence exists to support this contention at present. Furthermore, the roles of these molecules may differ according to the disease model and timing.

 Another player is ASK1, which selectively activates JNK and p38 signaling in response to a variety of stimuli, including ROS and cytokines [123]. The role of ASK1 in hepatocarcinogenesis has been investigated using the DEN-induced HCC model [54]. The number of tumors in *ASK*1^−/−^ mice is three times higher than that in wild-type controls, and cancer cell apoptosis is significantly suppressed in *ASK*1^−/−^ HCC. JNK activation and hyperphosphorylation of BimEL, which are required for death receptor-mediated apoptosis, including TNF-R and Fas, are attenuated in *ASK*1^−/−^ HCC but not in non-tumor tissues, suggesting escape from the antitumor immune response. In contrast, tumor-promoting functions of JNK, such as cell proliferation and neovascularization, are preserved in *ASK*1^−/−^ mice. Therefore, ASK1 is considered to play a major part in the tumor-suppressing role of JNK in hepatocarcinogenesis. In addition, activation of the p38 pathway is also attenuated in *ASK*1^−/−^ mice, and DNA damage-induced p21 upregulation via the p38 pathway is inhibited. Thus, ASK1 controls the tumor-suppressing function of stress-activated MAPK signaling through induction of apoptosis and the DNA damage response, and acts as a tumor suppressor in hepatocarcinogenesis (Figure 4). Furthermore, another recent study indicated that ASK1 and Bim are also required for sorafenib-induced apoptosis in HCC cells [124]. Therefore, ASK1 may play important roles in not only hepatocarcinogenesis but also in the therapeutic effects of anticancer drugs.

 In some cases, MAP3K may be a better therapeutic target than MAPK. To date, more than ten molecules have been identified as MAP3K in stress-activated MAPK signaling, which is much more than the number of the downstream MAPK (Figure 1) [125]. This fact indicates that the MAP3Ks are activated by various stimuli, and function as signaling hubs, integrating these signals into a unique pattern of MAPK activation, and eventually leading to a specific function of MAPK to the stimulus. Thus, blocking MAP3K may be able to inhibit specific function of MAPK. In addition, inhibition of JNK or p38 causes compensatory activation of another stress-activated MAPK, whereas inhibition of some MAP3Ks such as ASK1 does not cause such compensation, which may be advantageous in the clinical settings as discussed in the next section. However, which MAP3K is important in growth factor-mediated JNK activation in HCC remains unclear, especially *in vivo*, although MEKK1 and MLK3 have been reported to be involved in this role of JNK in non-HCC cells such as colon cancer [126, 127]. Therefore, further understanding of the regulatory system of stress-activated MAPK signaling by MAP3Ks is important.

## 5. Future Perspectives

 These recent studies have gradually clarified the roles and regulation systems of stress-activated MAPK in hepatocarcinogenesis, but some issues remain to be elucidated. One issue is that the role of these molecules may change according to the disease model. A good example is NF-*κ*B. As mentioned above, DEN-induced HCC is increased in hepatocyte-specific IKK*β* knockout mice and spontaneous HCC development is seen in hepatocyte-specific NEMO knockout mice, suggesting a role of NF-*κ*B as a tumor suppressor [69, 78]. However, experiments crossing transgenic mice expressing a nondegradable I*κ*B*α* mutant with MDR2 knockout mice, which show chronic inflammation in the portal area and subsequent cancer development, indicated a tumor-promoting role of NF-*κ*B [128]. These results indicate the dual roles of NF-*κ*B in hepatocarcinogenesis depending on the disease model. Thus, more appropriate models mimicking human chronic hepatitis and hepatocarcinogenesis are required. In this sense, TAK1 knockout mice may be a promising candidate because TAK1-deficient HCC develops in the context of chronic inflammation and fibrosis, as commonly found in human HCC [82, 83]. Furthermore, the time manipulation is also an important problem. For example, studies in TAK1 knockout and ASK1 knockout mice confirmed that too much apoptosis in the chronic inflammatory phase facilitates hepatocarcinogenesis, whereas too little apoptosis in already initiated cells also aggravates cancer progression, suggesting that the apoptotic function plays dual roles in a stage-dependent manner [54, 82, 83]. Therefore, therapeutic targets may need to be redefined according to the carcinogenic stage.

 The critical role of stress-activated MAPK signaling in hepatocarcinogenesis prompts their introduction (especially JNK1) into the clinical settings as therapeutic targets. Several inhibitors targeting JNK or p38 have entered clinical trials for other diseases. In particular, p38 inhibitors for inflammatory diseases, such as rheumatoid arthritis and inflammatory bowel disease, have been studied extensively [129, 130]. However, few have progressed beyond phase I clinical trials and none have yet appeared in clinical settings. One reason for this is the occurrence of side effects, such as liver toxicity. Although whether these are due to target inhibition or to the effects of the drugs on additional targets is unclear, cross talk among signaling pathways is considered to be one explanation because p38 inhibition induces compensatory JNK activation. Therefore, alternative strategies, such as the targeting of particular MAPK isoforms, including JNK1, upstream regulators, including MAP3K, and downstream effectors, including Bcl-2 family members, will be more beneficial than targeting the whole pathway. Note that the roles of these molecules differ among cell types, such as hepatocytes and myeloid cells, including Kupffer cells. Therefore, one must take into account its total function in disease progression. Furthermore, combination therapies are likely to be more important in the future. Hence, further understanding of these pathways will help to develop novel treatment strategies for advanced HCC or prevention of HCC development.

## 6. Conclusion

 Recent studies have indicated that MAPK signaling pathways play key roles and have potential as therapeutic targets in liver injury and hepatocarcinogenesis. Among them, JNK signaling is a promising therapeutic target. However, these pathways have a wide range of functions and show complex cross talk. In addition, their roles differ between cell types and disease processes. Our final goal is to establish them as new diagnostic markers and targets for therapies or prevention of liver diseases. In fact, recent report showed that activation of JNK in noncancerous liver tissue predicted a high risk of HCC recurrence after surgical resection, suggesting that testing JNK activation in the liver might be useful for the risk assessment of HCC occurrence or recurrence [131]. Thus, further understanding of these very important and complicated pathways, including surrounding modulators, is essential for application in clinical settings.

## Abbreviations

ASK1: Apoptosis signal-regulating kinase 1
DEN: Diethylnitrosamine
GalN: Galactosamine
HBV: Hepatitis B virus
HCC: Hepatocellular carcinoma
HCV: Hepatitis C virus
JNK: c-Jun NH_2_-terminal kinase
LPS: Lipopolysaccharide
MAPK: Mitogen-activated protein kinase
MAP3K: Mitogen-activated protein kinase kinase kinase
NASH: Nonalcoholic steatohepatitis
NF-*κ*B: Nuclear factor-kappa B
OIS: Oncogene-induced senescence
ROS: Reactive oxygen species
TAK1: Transforming growth factor *β*-activated kinase 1
TNF-*α*: Tumor necrosis factor-*α*
TNF-R1: TNF-receptor 1
Trx: Thioredoxin.
